# Genome-wide screening for genes whose deletions confer sensitivity to mutagenic purine base analogs in yeast

**DOI:** 10.1186/1471-2156-6-31

**Published:** 2005-06-02

**Authors:** Elena I Stepchenkova, Stanislav G Kozmin, Vladimir V Alenin, Youri I Pavlov

**Affiliations:** 1 Department of Genetics, Sankt-Petersburg State University, Sankt-Petersburg, 199034, Russia; 2 Laboratory of Molecular Genetics, National Institute of Environmental Health Sciences, RTP, NC 27709, USA; 3 Eppley Institute for Research in Cancer and Allied Diseases, the Department of Biochemistry and Molecular Biology, and the Department of Pathology and Microbiology, University of Nebraska Medical Center, Omaha, NE 68198, USA

## Abstract

**Background:**

*N*-hydroxylated base analogs, such as 6-hydroxylaminopurine (HAP) and 2-amino-6-hydroxylaminopurine (AHA), are strong mutagens in various organisms due to their ambiguous base-pairing properties. The systems protecting cells from HAP and related noncanonical purines in *Escherichia coli *include specialized deoxyribonucleoside triphosphatase RdgB, DNA repair endonuclease V, and a molybdenum cofactor-dependent system. Fewer HAP-detoxification systems have been identified in yeast *Saccharomyces cerevisiae *and other eukaryotes. Cellular systems protecting from AHA are unknown. In the present study, we performed a genome-wide search for genes whose deletions confer sensitivity to HAP and AHA in yeast.

**Results:**

We screened the library of yeast deletion mutants for sensitivity to the toxic and mutagenic action of HAP and AHA. We identified novel genes involved in the genetic control of base analogs sensitivity, including genes controlling purine metabolism, cytoskeleton organization, and amino acid metabolism.

**Conclusion:**

We developed a method for screening the yeast deletion library for sensitivity to the mutagenic and toxic action of base analogs and identified 16 novel genes controlling pathways of protection from HAP. Three of them also protect from AHA.

## Background

The accurate replication and repair of genetic material, which is a prerequisite for normal functioning of the eukaryotic genome and the prevention of cancer, relies on coordinated and faithful DNA synthesis. One important mechanism that ensures a high fidelity of DNA replication is a "cleansing" of the DNA precursor pool from deoxyribonucleoside triphosphates containing a modified base [1-4]. Such modified bases may have ambiguous base-pairing properties that will result in a high mutagenic activity after their incorporation into DNA during replication. A classic example of the detoxification mechanism is the elimination of dUTP and 8-oxo-dGTP from the dNTP pool by the *E. coli *dUTPase and MutT proteins, respectively [1,5].

**Figure 1:**
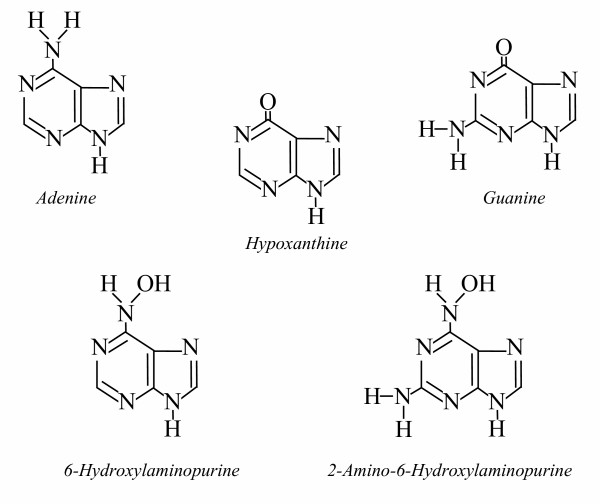
Chemical structures of HAP and AHA and natural purine bases.

Purine analogs 6-hydroxyaminopurine (HAP) and 2-amino-HAP (AHA) are powerful mutagens in bacteria, yeast, and higher eukaryotes [6,7]. It has been suggested that HAP-deoxyriboside-triphosphate (dHAPTP) is a possible endogenous contaminant of nucleotide pools under peroxyl radical stress [8]. HAP and AHA closely resemble the natural purines, hypoxanthine and xanthine (Fig. 1), and therefore, could be exploited to investigate the mechanism preventing mutations that are caused by non-canonical purine nucleotides [9-11].

It was proposed that purine salvage enzymes convert base analogs to the corresponding deoxyribonucleoside triphosphates, which are misincorporated or misreplicated during DNA synthesis, resulting in induction of mutations [12,13]. HAP-induced mutagenesis in yeast is elevated in strains with defects in proofreading activity of replicative DNA polymerases [14,15] and does not depend on excision, mutagenic recombination, and mismatch repair systems [14-16]. We have described several systems protecting cells from the mutagenic and inhibitory effects of HAP (see review [16]). One is the novel molybdenum cofactor-dependent system in *E. coli *[17]. It has yet to be determined if a similar system exists in higher eukaryotes. Another, versatile HAP-detoxification pathway relies upon the action of triphosphatase, Ham1p, which hydrolyze HAP-containing ribo- and deoxyribo-nucleotides to nucleoside monophosphates, and which prevent incorporation of base analog into DNA and RNA. We initially described the elevated sensitivity to HAP in yeast due to mutations in the *HAM1 *gene [18]. When we cloned and sequenced the *HAM1 *gene, we found that it has homologs in many organisms, from bacteria to humans [13], and proposed that the gene might code for new triphosphatase [16]. Then, the crystal structure of the Ham1p homologue from a thermostable bacterium (protein Mj0226) was determined [19]. It was found that the Ham1p ternary structure has common features with MutT. Homologs of the yeast Ham1p from other organisms possessed triphosphatase activity on dITP, ITP, XTP, and dHAPTP substrates (Kozmin and Pavlov, unpublished; Burgis and Cunnigham, personal communication; and [19-21]).

There are additional, less thoroughly studied, factors modulating purine base analogues mutagenesis in yeast (see [16] for review). For example, *aah1 *mutants are sensitive to HAP, suggesting that adenine deaminase Aah1p may deaminate HAP base to hypoxanthine [16].

In the present study, we carried out a genome-wide search for HAP and AHA sensitive mutants. The release of several complete sets of deletion mutants by the Yeast Deletion Project provides a powerful approach for different types of genome screens in yeast [22]. Haploid and diploid strains have already been used to detect new genes controlling sensitivity to different agents such as UV, ionising radiation, iron, and methyl methane sulfonate (MMS) [23-27], as well as spontaneous mutability [28]. This approach, when combined with other genomics approaches, helps to establish the biological functions of uncharacterized ORFs in yeast, many of which have human orthologs. This approach also allows us to decipher the network responses to endogenous and environmental stress [29]. The present study is the first systematic, genome-wide search for the mutations conferring sensitivity to mutagenic purine base analogs.

## Results

### Development of the screening method

**Figure 2:**
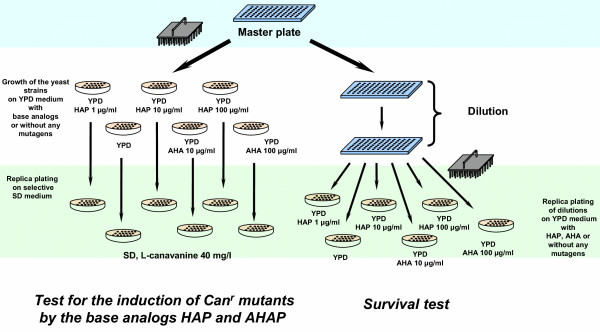
Scheme of the protocol for screening the yeast deletions library for base analog sensitivity and induced mutagenesis.

To develop a useful method for searching the yeast mutants sensitive to base analogs, we calibrated the experimental conditions using the wild-type strain, BY4742, and two previously described HAP-sensitive mutants, *ham1 *and *aah1 *(see [6,16]), created on BY4742 background. As shown in Fig. 2 and described in Materials and Methods, yeast were grown in a 96-well microtiter plate and then transferred, using a multiprong replicator device, to YPD plates containing base analogs. An induction of the Can^r ^mutants was monitored by replica-plating to the minimal-medium plates containing L-canavanine.

**Figure 3:**
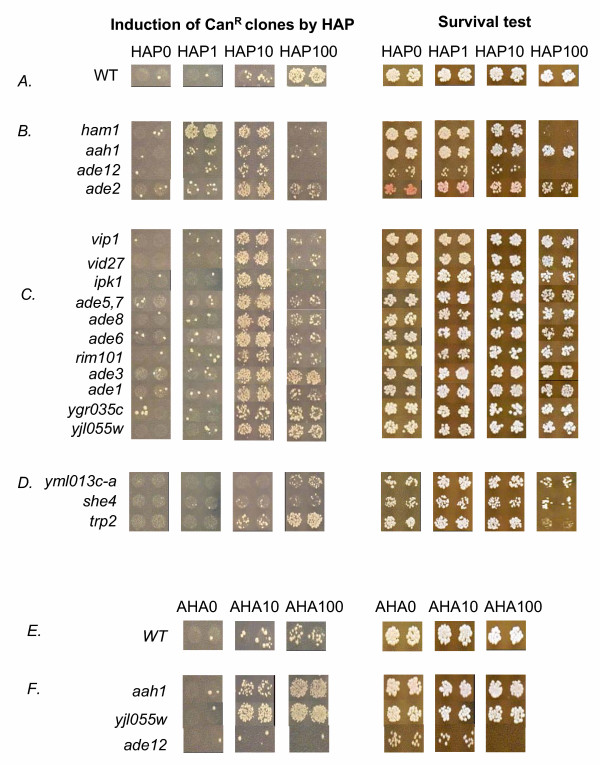
**Results of the screening of the yeast deletion library for elevated mutagenesis and sensitivity in micro-titer plates**. Left panel – Mutagenesis on selective plates with canavanine. Right panel – The estimation of the number of colony-forming units on YPD medium.

The results are presented in Fig. 3. The left panel of Fig. 3 shows the induction of canavanine-resistant mutants by HAP and AHA; and the right panel represents the survival of the tested strains on YPD plates in the presence of base analogs. In the wild-type strain, as in the *ham1 *and *aah1 *mutants, 1–3 spontaneous canavanine-resistant colonies per spot arise in the absence of mutagen (Fig. 3A and 3B, left panel). In our experimental conditions, 1 μg/ml of HAP did not induce Can^r ^mutants in the wild-type strain. A moderate induction of Can^r ^clones (fewer than ten per spot) was observed at 10 μg/ml of HAP and a very strong HAP-induced mutagenesis was observed in the wild-type strain at 100 μg/ml of HAP (Fig. 3A, left panel). For comparison, 100 μg/ml of AHA were only moderately mutagenic (compare Fig. 3A and Fig. 3E, left panel). Furthermore, both HAP and AHA did not affect the viability of the wild-type strain, even at the maximal concentration of 100 μg/ml (Fig. 3A and 3E, right panel).

In the *ham1 *strain, 1 μg/ml of HAP induced Can^r ^mutants with the similar frequency that was observed in the wild-type strain at 2 orders in magnitude higher concentration of analog, 100 μg/ml (compare Fig. 3A and 3B, left panel, phenotype of hypermutability, HM). The hypersensitivity of the *ham1 *mutant to the toxic action of HAP was clearly detectable at 100 μg/ml of HAP (Fig. 3B, right panel). We will refer later to this phenotype as hypersensitivity, HS. Note that a reduction of the number of canavanine-resistant clones at 10 and 100 μg/ml of HAP, in comparison with 1 μg/ml of HAP observed in the *ham1 *mutant (Fig. 3B, left panel, another manifestation of hypersensitivity phenotype, HS), is also due to a dramatic decrease of survival. When the *aah1 *mutant was tested (Fig. 3B, second row, for HAP and Fig. 3F, first row, for AHA), the drop of viability was less severe than that for the *ham1 *mutant (phenotype of elevated sensitivity, S). HAP-induced mutagenesis was detectable at a low dose of 1 μg/ml, but was much less compared to the *ham1 *mutant (phenotype of elevated mutability, M). Mutagenesis was somewhat stronger at the dose 10 μg/ml and was not seen at 100 μg/ml (another manifestation of elevated sensitivity, S). For the *aah1 *mutant, AHA-induced mutagenesis was moderate at the dose of 10 μg/ml and very strong at a concentration of 100 μg/ml. The *aah1 *mutant exemplified what we expect to observe for mutants moderately sensitive to both HAP and AHA. These data suggested that the procedure we devised for micro-titre plate format is effective for the detection of mutants with altered parameters of HAP and AHA sensitivity and mutability.

### Screening of the deletion strains library

We screened the yeast deletion strains library as described above. After an initial screening of 4,823 deletion strains, 43 mutants strains appeared to affect base analog-induced killing or mutagenesis. However, in subsequent testing of candidate strains in same type of plate tests, we have confirmed HAP-sensitivity of 16 mutants (Fig. 3, Table 1, column 4, where we refer to phenotypes of mutants according to abbreviations described in previous section). Next, we examined the mutability and survival of these 16 mutants in quantitative tests with HAP (see Materials and Methods). Based on the results of these two types of tests, we categorize HAP-sensitive strains in three groups, as shown in Table 1. Group I comprises, in addition to *ham1 *and *aah1 *strains, mutants *ade12 *and *ade2*. These strains were hypersensitive to the mutagenic and lethal action of HAP in both types of tests (Fig. 3 and Table 1, columns 4–6). Strains of this group were sensitive to the low doses (1 and/or 10 μg/ml) of HAP, in contrast to the wild-type strain. The *ade12 *mutant was almost as mutable as the *ham1 *strain, but the hypermutability could only be demonstrated in a quantitative test, due to poor plating efficiency (compare Fig 3B, row 3 and Table 1, column 5, row 3). The *ade2 *mutant was as mutable as the *aah1 *mutant (Table 1). Deletion mutants of the first group showed variable degree of sensitivity to HAP-induced killing Table 1, column 6).

**Table 1:** HAP-sensitivity of the mutants selected in our screening.

Class of mutants	Gene or ORF name deleted	Functional group	Response HAP in spot tests^†^	Induced mutant frequency (×10^-7^) by HAP^#^	Survival in presence of HAP^#^
Wild type			-	400	100%

Class I: mutants hypersensitive to HAP	*HAM1*	DNA^1^	HM, HS	13000	17%
	ADE12*	DNA	HM, HS	10000	30%
	AAH1*	DNA	M, S	5500	30%
	*ADE2*	DNA	M, S	4400	70%

II class: Mutants sensitive to mutagenic effect of HAP	*VIP1*	Cell^2^	M, S	840	76%
	*VID27*	Metabolic^3^	M, S	600^°^	82%
	*IPK1*	Metabolic	M, S	1400	100%
	*ADE5,7*	DNA	M	1900	100%
	*ADE8*	DNA	M	1500	100%
	*ADE6*	DNA	M	1100	100%
	*RIM101*	meiosis	M	1100	100%
	*ADE3*	DNA	M	940	100%
	*ADE1*	DNA	M	860	100%
	*YGR035c*	unknown	M	500^°^	100%
	Yjl055w*	Metabolic	M	500^°^	100%

Class III: Mutants sensitive to killing	*YMl013c-a*	Unknown	S	230^°^	60%
	*SHE4*	Cell	S	600^°^	30%
	*TRP2*	Metabolic	HS	250^°^	60%

^†- ^HM – hypermutable, M – more mutable than wild-type, HS – hypersensitive, S – more sensitive than wild-type, see first paragraph od the Results Section for the explanation.

^# ^– 25 μg/ml

^1 ^– 'DNA' – includes genes involved in the control of DNA precursor metabolism, purine salvage, DNA repair.

^2 ^– 'Cell' – includes genes involved in cytoskeleton organization, cell walls and organelles.

^3 ^– 'Metabolic' – includes genes involved in general metabolic pathways.

* – These mutants were also sensitive to the mutagenic or toxic effect of AHA (see Fig. 3).

^° ^– small difference from the wild-type strain was reproducible and statistically significant by Wilcoxon-Mann-Whitney test.

Eleven mutants fall into Group II. Mutants of this class were sensitive to the mutagenic effect of HAP, but their growth was not severely inhibited on HAP-containing medium. As a result, there is a smaller difference, in comparison to mutants of the group I, or no difference in the number of Can^r ^mutants induced by 10 and 100 μg/ml of HAP in spot-tests (Fig. 3C, Table 1, column 4). These strains produced some HAP-induced Can^r ^mutants at 10 μg/ml of HAP, whereas the parental strain was non-mutable at this HAP concentration. We also did not detect any substantial drops of viability after growth in liquid media containing 25 μg/ml of HAP (Table 1, column 6). Group II was not homogeneous in respect to HAP mutability and sensitivity. The six most sensitive mutants in the group are *vip1, vid27, ade1, ade5,7, ade6, ade8, ipk1 *and *rim101 *(see Table 1). These mutants showed a decrease in the number of Can^r ^mutants in a qualitative test when concentration of HAP increased ten-fold (up to 100 μg/ml), which is an indication of some cell death at very high doses of HAP (Fig. 3C). Mutants *yjl055w*, *ygr035c*, and *ade3 *were more resistant to HAP, since the number of Can^r ^colonies was similar at 10 and 100 μg/ml of HAP. As could be seen from the results of quantitative assay, mutant classification is quite conditional and there is substantial variation in responses between mutants of the Group II, but all of them were more mutable that the wild-type strain.

Finally, Group III includes mutants *she4, trp2*, and *yml013c-a; *which were sensitive to HAP-induced killing, but not to HAP-induced mutagenesis (Fig. 3D and Table 1, column 6). In quantitative tests the *yml013c-a *and *trp2 *mutants were even less sensitive to the mutagenic action of HAP than the wild-type strain (Table 1). The *she2 *mutant was slightly more mutable that the wild-type strain only in quantitative test. The existence of such a type of mutants suggests that the toxic effect of HAP in yeast may be not only due to the induction of lethal mutations, but also due to inhibition of certain metabolic pathways.

In the present study, we have also characterized three AHA-sensitive mutants, *aah1*, *ade12*, and *yjl055w *(Fig. 3E and 3F). Remarkably, all of these mutants were also sensitive to HAP (Fig. 3C and Table 1). Two of those mutants, *aah1 *and *yjl055w*, were AHA-hypermutable; whereas a*de12 *strain did not mutate in the presence of AHA, but was sensitive to AHA-induced killing. In a quantitative test, 50 μg/ml of AHA did not inhibit survival of the wild-type strain, *aah1 *and *yjl055w *strains; whereas survival of the *ade12 *mutant was reduced to 50%. In the *aah1 *and *yjl055w *strains, 50 μg/ml of AHA induced canavanine-resistant mutants with the frequencies 120 × 10^-7 ^and 270 × 10^-7^, respectively, that was 4–9-folds higher than observed in the wild-type and *ade12 *strains (30 × 10^-7^, in both strains).

## Discussion

In this study, we elaborated the technology for testing base analog-induced mutability and killing of thousands of yeast strains in microtiter format (see Fig. 2). We found that the method is quite sensitive and reliable. Next, we screened the library of haploid yeast strains carrying deletions in all nonessential ORFs for the sensitivity to mutagenic base analogs HAP and AHA. We have found 16 novel HAP-sensitive mutants that fall into several groups, based on the sensitivity profiles (Fig. 3 and Table 1). One group comprises the mutants that are HAP-hypermutable and grow poorly in the presence of HAP. Another class comprises strains with elevated HAP-mutability that grow normally on medium containing HAP. Finally, the third group includes the mutants sensitive to HAP-induced killing, but not to HAP-induced mutagenesis. We have also isolated three AHA-sensitive mutants. All of them were HAP sensitive as well. We summarized the properties of the genes found in our screening in Additional file 1.

One interesting result from our study is that none of the genes involved in the control of base analogs sensitivity except two genes, *YML013c-*a and *ade12*, were found in screenings for genes controlling sensitivity to the other types of mutagens, MMS, UV, and ionizing radiation [23-27] or for elevated spontaneous mutagenesis [28]. It was previously reported that deletion of the *YML013c-a *and *ADE12 *open reading frame specifically enhanced sensitivity to killing (as in case of HAP) induced by γ-radiation and bleomycin, but did not affect sensitivity to MMS, UV, and hydroxyurea [23,30]. It is known that there are overlaps of the sets of genes detected in genome screenings for the MMS-, UV- or ionizing radiation-sensitive strains [29]. Usually, those spectra include genes controlling DNA replication, recombination, and DNA repair. In our study, we did not find any of those genes. This is consistent with our earlier observation that the mutagenic action of HAP in *S. cerevisiae *does not depend on nucleotide excision repair, mutagenic repair, and mismatch repair [16]. A system to repair non-canonical purines and, probably HAP and AHA, in DNA has been recently characterized in *E. coli *[9,10]. It is possible that yeast *S. cerevisiae *lacks such a repair system.

**Figure 4:**
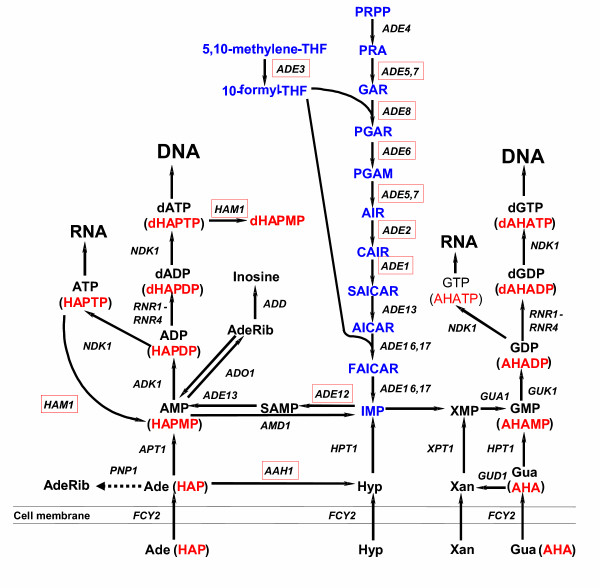
**Purine salvage and purine biosynthesis *de novo *in yeast *Saccharomyces cerevisiae***. Intermediates of the purine biosynthesis *de novo *are designated in blue. The salvage pathway is presented in black. Genes, whose deletions lead to HAP and/or AHA sensitivity are highlighted by red boxes. The proposed conversions of HAP and AHA are represented in brackets below the adenine and guanine metabolites, respectively. Dashed arrows represent hypothetical pathways that were not demonstrated experimentally for a given substrate. Abbreviations:Purine biosynthesis *de novo*: PRPP – 5-phospho-ribosyl-1α-pyrophosphate, PRA – 5-phospho-β-D-ribosylamine, GAR – 5-phosphoribosylglycinamide, FGAR – 5'-phosphoribosyl-*N*-formyl glycinamide, FGAM – 5'-phosphoribosyl-*N*-formylglycinamidine, AIR – 5'-phosphoribosyl-5-aminoimidazole, CAIR – 5'-phosphoribosyl-5-aminoimidazole-4-carboxylate, SAICAR – 5'-phosphoribosyl-4-(*N*-succinocarboxamide)-5-aminoimidazole, AICAR – 5'-phosphoribosyl-4-carboxamide-5-aminoimidazole, FAICAR – 5'-phosphoribosyl-4-carboxamide-5-formamidoimidazole, SAMP – adenylosuccinate, 5,10-methylene-THF – 5,10-methylenetetrahydrofolate, 10-formyl-THF – 10-formyltetrahydrofolate. Salvage: Ade – adenine, AdeRib – adenosine, Hyp – hypoxanthine, Gua – guanine, Xan – xanthine.

Based on our data, we propose that, in yeast, the major base analogs protective mechanism is a control of the quality of DNA precursor pool that prevents incorporation of base analogs into DNA. This mechanism may work at several levels: transport of analogs into cells, detoxification of analogs by metabolic enzymes, maintenance of nucleotide pools, and fidelity control of DNA replication (Fig. 4). HAP and AHA are likely transported into the yeast cell by the same permeases, which are involved in transport of natural purines. One candidate is purine-cytosine permease, Fcy2p, a major purine (adenine, guanine, and hypoxanthine) and cytosine transporter in yeast [31]. According to our unpublished data, *fcy2 *mutants are resistant to HAP. Thus, the active transport of HAP is the first critical step in the HAP mutagenic pathway. The next step is a conversion of the base analog to the corresponding ribonucleoside monophosphate by enzymes of the purine salvage pathway. Previously we observed that the inactivation of the *APT1 *gene, encoding adenine phosphoribosyl transferase, led to a severe decrease of the mutagenic effect of HAP [16], suggesting that this enzyme plays a key role in the biosynthesis of HAP-riboside-5'-monophosphate (HAPMP). HAPMP then may be converted to the corresponding nucleoside triphosphate, which could be ambiguously incorporated into DNA by DNA polymerases and provoke replication errors in the subsequent replication cycles [13].

The mechanism preventing HAP- and AHA-induced toxicity most likely involves the conversion of base analogs to non-mutagenic metabolites by purine salvage enzymes. The main argument for this hypothesis is that HAP could be utilized by yeast cells as a sole purine source [13,18,32]. The first enzyme in this HAP and AHA detoxification pathway is adenine aminohydrolase, encoded by the *AAH1 *gene. Aah1p from several microorganisms has been biochemically characterized (see [33]). Is has a broad substrate specificity and is capable of converting both adenine and its six-substituted analogs into hypoxanthine *in vitro*. According to our data, yeast Aah1p may convert HAP to hypoxanthine [12] and AHA to guanine *in vivo*, since the inactivation of the *AAH1 *leads to a defect in this conversion that is readily detected by UV spectroscopy of yeast medium (unpublished observation). In the *aah1 *mutant, the base analog intracellular concentration increases. We propose that this causes the elevation of base analog-induced mutagenesis (Fig. 3B and 3E, Table 1).

Interestingly, in our screening we did not detect the *amd1 *mutant, deficient in AMP aminohydrolase, that catalyze deamination of AMP to IMP [34]. We disrupted this gene by the *kanMX *cassette in several yeast strains and did not see any effect on HAP-induced mutagenesis anywhere. Thus, deamination of HAP at the mononucleotide level does not play an important role in HAP detoxification. This can be due to several reasons: the inability of the Amd1p to hydrolyse HAPMP, the minor role of the *AMD1 *gene in yeast, or the short life-time of the HAPMP in yeast cells.

We have found that inactivation of adenilosuccinate synthase (ASS or Ade12p) encoded by the *ADE12 *gene strongly enhanced HAP-induced mutagenesis and AHA-induced killing (see Fig. 3B and 3F, and Table 1). The primary function of this enzyme is the conversion of IMP to SAMP in the pathway of AMP biosynthesis *de novo *(Fig. 4). We propose that the reason for this hypersensitivity is the dysregulation of purine biosynthesis, as follows. First, the blocking of this step of purine biosynthesis causes accumulation of IMP in the cell. The excess of IMP is probably toxic for the cell, since *ade12 *mutants have a slow-growth phenotype that can be rescued by blocks of the earlier steps of the *de novo *purine biosynthesis pathway (Dr. A. M. Zekhnov {St-Petersburg State University}, personal communication). Accumulated IMP can be phosphorylated to ITP by nucleotide phosporylases. Thus, we propose that, in the *ade12 *mutant, an excess of ITP may saturate Ham1p triphosphatase, an essential enzyme for the destruction of HAPTP and dHAPTP, which leads to increased HAP-sensitivity (see Fig. 3). The data obtained recently in bacteria are consistent with this hypothesis. It was shown that, *in vitro*, *E. coli *Ham1p homologues protein, RdgB, is a triphosphatase that acts to hydrolize non-canonical DNA precursors, dIPT and dXTP. The Ham1p protein was shown to possess a similar activity on dITP, dXTP, and dHAPTP substrates (Kozmin and Pavlov, unpublished; Burgis and Cunnigham, personal communication; and see refs. [19-21]). In *E. coli*, the *rdgB *mutation is synthetically lethal with *recA*. As proposed, absence of RdgB leads to a dramatic increase of hypoxanthine and xanthine in DNA. Accordingly, base excision repair of such modified bases occurring in opposite strands may generate double strand breaks that require the RecA function to be repaired. However, over-expression of adenilosuccinate synthase PurA (homolog of yeast Ade12p) suppresses this lethality [9]. This suggests a critical role of ASS in the regulation of intracellular concentration of genotoxic hypoxanthine-containing nucleotides.

We found that certain mutations affecting IMP biosynthesis *de novo *enhance HAP-induced mutagenesis (Table 1). Seven of sixteen newly identified genes controlling HAP and AHA sensitivity are involved in AMP biosynthesis (see Additional file 1 and Fig. 3). It is known that the regulation of the AMP biosynthesis pathway by adenine is mediated by SAICAR, one of the precursors in adenine biosynthesis *de novo *[35]. The accumulation of certain purine biosynthesis by-products may play a role in the regulation of the nucleotide pool. A defect in endogenous purine biosynthesis probably alters nucleotide pools to favour dHAPTP mis-incorporation into DNA or leads to HAP toxicity. In respect to this hypothesis, it is important that there is a difference in the level of HAP-induced mutagenesis among the strains carrying mutations in the genes of AMP biosynthesis. The less sensitive mutant is *ade3 *(Fig. 3 and Table 1). The *ADE3 *does not directly control any steps of purine biosynthesis. It encodes the C1-tetrahydrofolate synthase that provides C1-tetrahydrofolate, an indispensable precursor for AMP, histidine, thymidylate, and methionine biosynthesis (see Fig. 4). In this respect it is of interest that *ade4, ade16 *and *ade17 *mutants, also defective in IMP biosynthesis, were not found in our screen and were not sensitive to HAP or AHA when constructed *de novo *and tested directly (data not shown). The *ADE4 *stands apart because PRPP, a substrate of the product of the *ADE4 *gene, serves as a precursor for additional biosynthetic pathways. This prevents by-product accumulation and might be one of the explanations of lack of HAP sensitivity of the *ade4 *mutants. The single *ade16 *and *ade17 *mutants also do not lead to byproduct accumulation because *ADE16 *and *ADE17 *are isozymes and the inactivation of one gene does not block the pathway.

We have found that 6 genes detected in our screening, *VIP1, VID27, IPK1, YGR035c, YML013c-a *and *SHE4*, are putatively involved in cell organization or genetically interact with cell-cycle control genes (see Additional file 1). This observation provides new perspectives on the mechanisms of base analogs-induced mutagenesis. It is possible that there is a specific structural route, mediated by cell cycle and cytoskeleton components, initiated by penetration of the analog inside the cell to its final target. We have also identified several hypothetical genes critical for the HAP and/or AHA resistance. This may be an initial clue to their functional significance.

Finally, we would like to mention that out of the 18 genes we found to be involved in HAP and AHA sensitivity control, 11 (60%) have orthologs in all groups of organisms, including mammals. Therefore, the results have relevance to higher eukaryotes and humans as well (see Additional file 1).

## Conclusion

We identified novel mutants sensitive to mutagenic and toxic effects of purine base analogs. AHA sensitivity was not previously described for three of the mutants identified in this study. The results reveal a complex control of base analogue mutagenesis by genes encoding the components of metabolic pathways and cytoskeleton.

## Methods

### Yeast strains and media

We have used a set of 4,823 *S. cerevisiae *mutants carrying deletions of all non-essential ORFs created in the haploid strain BY4742 (*MATα his3Δleu2Δlys2Δura3Δ*). The information about the deletion strains set is available from the Yeast Deletion Project site: . Deletion strains were constructed by replacement of the ORF's with the *kanMX4 *cassette, which confers resistance to geneticin [36].

The standard yeast complete media (YPD) and minimal synthetic media (SD) were used [37]. Deletion strains were cultivated on YPD medium, supplemented with 200 μg/ml of geneticin. Sensitivity to HAP and AHA was examined on YPD media containing analogs in concentrations 100 μg/ml, 10 μg/ml and 1 μg/ml. SD medium containing 40 μg/ml of L-canavanine was used for the selection of Can^r ^mutants.

### Base analogs sensitivity tests

HAP was purchased from MP Biomedicals (Irvine, California, USA). AHA was custom- synthesized by Dr. I. Kuchuk at the Department of Chemistry of Indiana University (Bloomington, Indiana, USA) by the method of Janion [38]. Both chemicals were dissolved in DMSO (Fisher, USA) with mild heating. A single colony of each tested strain was inoculated into a well of 96-well microtitre plate containing 200 μl of liquid YPD medium (see Fig. 2). Microtiter plates were incubated for 2 days at 30°C with agitation, to reach a stationary phase (approximately 10^8 ^cells/ml). For mutagenesis assay, cells were then plated by a multiprong replicator device (approximately 5 μl of cell suspension per prong) to the YPD plates containing various concentrations of HAP or AHA, as shown on Fig. 2. After one day of incubation, the plates were replica-plated on SD minimal-medium plates containing L-canavanine. The plates were incubated 5 days and inspected for induction of Can^r ^mutants.

For the survival test, cell cultures from the microplates were diluted in water in series of 96-well microplates, using a multichanel pipette (see Fig. 2). Diluted cells suspensions were plated to YPD plates containing HAP or AHA by a multiprong replicator device. After 2–3 days of incubation, the number of colonies was recorded. Strains that produced smaller colonies or a smaller number of colonies on the HAP-containing medium, relatively to wild-type strain, were classified as sensitive to killing.

### Quantitative assay of the base analog-induced mutagenesis

For each strain and each concentration of base analogs to be tested, we prepared six independent cultures by inoculating a single colony into 1 ml of liquid YPD medium with or without mutagens. After two days incubation in the roller drum, the mutant frequencies were determined by plating of appropriately diluted cell suspensions on minimal-medium SD plates supplemented with L-canavanine (to determine the number of canavanine-resistant cells per culture), and on YPD plates (to obtain the total number of cells per culture). Then the frequency of mutants was calculated as described [14]. Each experiment was repeated at least three times. We have used several doses of HAP and found that in this type of test the most reproducible results are obtained at dose 25 μg/ml. The statistical significance of differences between variants was estimated by Wilcoxon-Mann-Whitney nonparametric criterion.

## Authors' contributions

Youri Pavlov and Elena Stepchenkova designed this study. Elena Stepchenkova performed the experimental work and wrote the initial draft of the manuscript. Vladimir Alenin, Youri Pavlov, Stanislav Kozmin, and Elena Stepchenkova analyzed the data set and wrote the final version of the paper.

## Supplementary Material

Additional File 1 Annotation of the genes whose deletion results in HAP and AHA sensitivity Click here for file
