# Supplementary material for: Genome-wide screening for genes whose deletions confer sensitivity to mutagenic purine base analogs in yeast
Source: BMC Genet. 2005 Jun 2;6:31. doi: 10.1186/1471-2156-6-31 (PMC1173102; doi:10.1186/1471-2156-6-31)
Supplement: Additional File 1 — Annotation of the genes whose deletion results in HAP and AHA sensitivity [file 1471-2156-6-31-S1.pdf]

Annotation of the genes whose deletion results in HAP and AHA sensitivity

Class of mutants	Gene name	Molecular Function	Biological process	Orthologs in other organisms*
Class I: Mutants sensitive to killing and mutagenic effect of HAP	*HAM1*	NPPT[1]ase for modified purines	NTP pools cleansing	all
	*ADE12***	Adenilosuccinate synthase	Biosynthesis of purine nucleotides	all
	*AAH1***	Adenine deaminase	Purine salvage	all
	ADE2	PRAIC[2]	Biosynthesis of purine nucleotides	all
Class II Mutants sensitive to mutagenic effect of HAP	*VIP1*	unknown	Cytoskeleton organization and biogenesis	all
	VID27	unknown	Involved in vacuole import and degradation	all
	IPK1	Inositol/ phospho-inositolkinase	Myo-inositol metabolism; lethal with the *bim1* (microtubule stabilizaton), *tof1* (topoisomerase interacting factor) and *chs1* (chitin synthase)	Fungi
	ADE5,7	AIRS_GARS[3]	Biosynthesis of purine nucleotides	all
	ADE8	PGFTase [4]	Biosynthesis of purine nucleotides	all

	ADE6	PRFGSase[5]	Biosynthesis of purine nucleotides	all
	RIM101	Putative transcription factor	unknown	Fungi
	ADE3	THPS[6]	Biosynthesis of purine nucleotides	all
	ADE1	PRAISCS[7]	Biosynthesis of purine nucleotides	all
	YGR035c	Potential Cdc28p substrate	unknown	None
	*YJL055w***	Lysine-decarboxylase like protein	unknown	Archea, Eubacteria, Fungi, Plants
Class III Mutants sensitive to killing	*YML013C-A*	unknown	unknown, inviable with the *cdc73* null	None
	SHE4	Protein containing a UCS (UNC-45/CRO1/SHE4) domain, binds to myosin motor domains to regulate myosin function	polarization of the actin cytoskeleton, and asymmetric mRNA localization	Fungi, Animals
	TRP2	anthranilate synthase	tryptophan biosyntesis	Archea, Eubacteria, Fungi, Plants

Table legend text.

All - Archea, Eubacteria, Fungi, Plants, Animals

* - any number of species with orthologs in the particular kingdom considered as positive

** - mutants sensitive to mutagenic or toxic effect of AHA

[1] - Abreviations: NPPT – nucleoside triphosphate pyrophosphatase

[2] – PRAIC - phosphoribosylamino-imidazole-carboxylase

[3]– AIRS_GARS - aminoimidazole ribotide synthetase, glycinamide ribotide synthetase

[4]- PGFT - Phosphoribosyl-glycinamide formyltransferase activity

[5]- PRFGS -5'-phosphoribosyl-formyl glycinamidine synthetase

[6] -THPS-tetrahydropholate synthase

[7] – PRAISCS -phosphoribosyl amino imidazolesuccinocarbozamide synthetase
